# Rev. Mr. Hill, on Vaccination

**Published:** 1807-04

**Authors:** Rowland Hill


### *To the Editors of the Medical and Physical Journal.*

GENTLEMEN,

IN the former paper I sent to your Journal, I ventured upon some observations respecting cutaneous diseases, that whatever they might be, they communicated no other disease excepting their own, but as the constitution might be weakened and injured thereby; and in this I find myself supported by gentlemen of the first respectability in the medical profession.

I mentioned also an instance of ONE *supposed* failure, not of my own, but of one inoculated at Surry Chapel School-room, as a station of the Jennerian Society. If the patience of the public be not nearly exhausted by the investigation of failures, I shall beg their attention to the following case, especially as it gives another strong specimen how much credit ought to be attached to the redoubtable Dr. Moseley, in what he has been pleased to advance within my personal knowledge, meaning it as a sample how far he may be worthy of credit, respecting a variety of other cases which he has produced, with a

 design design to alarm the public mind against this most beneficial discovery.

The case relates to the child of Frederick Batesford, in Brunswick Court. Dr. Moseley tells us, in his accustomed style, p. 211, that "the child after he had the cow-pox, by one of Rowland Hill's inoculators at Surry Chapel, was attacked with the small-pox about the 16th of April." Then he adds, this was in the month of April. Afterwards, in the month of May, at the annual meeting of the Jennerian Society, Rowland Hill said, as it is stated in the General Evening Post, already mentioned, "I solemnly declare before God that I have never failed in a single instance." So, that here I am accused by this man, of having told a wilful and deliberate untruth before the Duke of York, and several others of the first respectability and rank on that festal day.—Here then is the death-wound to the Doctor's character or mine.

I shall not shrink from the challenge, under the pitiful subterfuge that the Doctor shifts his ground in the same paragraph, that another was the inoculator, therefore the failure could not be charged on me; for I then said more than was published in that paper, "that neither from my own personal application of the lancet, nor from the list of those who had been inoculated at Surry Chapel, had I at that time met with one single failure." Now I can bring *the most incontestable proof*, that it was not before, but early in the week after the Jennerian dinner that Batesford brought her child to my door. I will not say, whether Dr. Moseley, and his brother *searcher*, Mr. Lipscomb, were incorrectly informed of the date of this event; but, in my life time, I never met with any thing that looks more like *design* or *art* in thus altering dates for purposes so ungenerous and base. But this is not the only falsehood stuffed into this single story. The Doctor says, "He, the child, was *very* ill during the eruptive fever." The parents declare the eruptive fever was slight and inconsiderable, not more than might be expected at the cutting of the teeth. The child, however, being indisposed, the mother washed it with warm water, and kept it confined and close at a season when the weather began to get warm also, and in a situation, as these *Gentlemen* well-know, the most impure, with a stagnated ditch before the door. Thus all circumstances were conjoined that might have rendered the disease confluent and mortal. The Doctor further says, "the child had a numerous crop of pustules". The parents asserted to him and Mr. Lipscomb, that he he had very few pustules indeed over the body, and by no means a heavy sprinkling over the face and limbs, and these of a light watery kind, quite different to what they had ever seen in the common small-pox; and, that they began to turn on the sixth day, and to dry off on the seventh, without leaving a single pit behind. But the Doctor exposes himself still further, as it respects his integrity in the same short story. Every person who means to deceive, does it either by the misrepresentation of truth, or by the suppression of facts. And here again the parents declared to these Doctors, that the child, who was under this slight infection of the small-pox, if it really was so, never had the cow-pock in a similar style to the brother; he had but one vesicle, and that vesicle was comparatively small, and the efflorescence very inconsiderable. Had these *Gentlemen* been possessed of a spark of candour, they would have determined that the child's constitution was not then capable of receiving the genuine disease to its proper extent, as they know is sometimes the case in their variolous inoculation.

The same disingenuous spirit prompted them to suppress other facts. They *knew* that all the children in that court, who were numerous, and who were vaccinated before, were brought into play with the child, while Mr. Ring, by my request, attended, and re-inoculated all the family from the infected child. Nay more than this—One woman, whose children were entirely unprotected, either by vaccination or by small-pox inoculation, with but one intervening house between them, brought those children into Batesford's house, to kiss and abide with the same supposed infected child; and in no way whatsoever was there a single instance of another being infected thereby.

Now, though all these circumstances may still create a doubt, whether this can be admitted as a real failure, though as to myself I am quite ready to admit it, yet it is to me a very corroborating proof of what many of the medical gentlemen have asserted, that where the cow-pox fails to protect, perhaps once in a thousand instances, it always disarms the small-pox of its fatal consequences, and renders it a mild and harmless disease. I have seen two instances of the same sort, though both of them milder still, who passed under the hands of other inoculators. Mr. Astley Cooper, whose eminence in his professional line is universally acknowledged, was so kind as to give me this hint to attend to this observation, as such supposed failures might occasionally occur. I have done so, and

 thank thank Dr. Moseley that he has given me another opportunity of quoting the case of Batesford's child, as a corroborating proof of this general remark. If on this ground only, the promoters of vaccination can establish the good effects of the discovery, its advocates are the greatest friends to the human race, and its opposers are as great enemies to the public good as is the horrid pestilence itself. When once a writer has been detected in deviating from the laws of truth, either by gross perversions or evident misrepresentations, we are ever suspecting what he may afterwards advance.

Another instance, similar to the former, as it respects his little attention to truth, appears in his last publication on page 210, where he thus exclaims. "It is shocking, Rowland, with what unfeeling levity you mention, p. 34, your having been charged with *killing* a poor woman's child, which died after your cow-pox inoculation! To believe that it died from any other cause, depends upon the credit which may be given to *your* assertion. You know my opinion on that point."

Hence it appears, first, that I can kill children by *cow-poxing* them, (a vulgar expression of his) when I am above a hundred miles distance from the place.

Secondly, that the cow-pox can kill people almost immediately after inoculation, before the disease could have taken any possible effect on the constitution. And,

Thirdly, that the Doctor has a right to say just what he likes, in opposition to those who are perfectly well acquainted with the case.

The fact is, as I before stated, that the child was almost immediately after inoculation, seized with a violent inflammation in the bowels, and died within three days of its first seizure. Mr. Gillham, in the presence of Mr. Warner, both of them members of the College of Surgeons, was allowed to open the body, and discovered that the inflammation was probably brought on by arsenick, which the parent acknowledges was very incautiously placed in the house. Now, who is to give credit to the *mere ravings* of such a man, who supposes he has a right to say any thing that comes uppermost and then to conclude that he has proved the point?

A similar sample we have in p. 224, where he is pleased to tell the public, that "I make not less, *as he is informed*, than *three thousand pounds* a year by nothing—by worse than nothing—by noise—by horrid Stentorian noise." Now, though I have an equal right to *rant* from the pulpit, pit, as he has from the press; yet, when the Doctor was about it, he might as well have told the public, that my gainings were annually thirty thousand, instead of three; and, though it is nothing to him, or any one else, that I gain by my *stentorian noise,* nor yet any thing to the present point in controversy; yet, if this Doctor will only make over to me all his gainings, which he turns in upon the score of his variolous inoculation, all my profits, as a preacher, shall be immediately at his service. I am almost ashamed to produce any further evidence to demonstrate what little credit is due to the rash assertions of this strange hero; I cannot resist, however, commenting upon another, as curious as any of the former.

Early in May last, soon after the publication of my little tract, Dr. Moseley sent me a note, from the style of which, I remember, it should rather have been dated from Bedlam, than from Albany House. Now I always treat insolence with the contempt it deserves. This Doctor, however, had the vanity to suppose I should answer his note, and that I should obey the summons he was pleased to send me, that I might be convinced of the mischief we *Cow-poxers* were guilty of in the family of a person of the name of Seyffert; but not thinking it worth my while to give myself the least trouble to investigate a case from such a reporter, especially as I was not the inoculator, he bursts out into the following ravings.

"You, Rowland Hill, have, for some years past, done ten times more mischief by your cow-pox inoculation than would have been occasioned in all England in the same space of time, by small-pox inoculation *conducted properly,*" an expression of notable import.

"When I wrote to you on the 3d of March last;" so this wonderful Doctor can read books six weeks, or I believe more, before they are printed, (but dates are of no consequence with him), to go to Little Chelsea, and see Mr. Seyffert's daughter, a case of small-pox after cow-pox, on the 6th day of eruption; what sullen fit assailed you, that you did not answer my letter?"

"The cow-pox brutalises the manners of men." So that all vaccinators, however respectable, are *brutalised,* and the Doctor is the *Gentleman,* and this is the *Gentleman* who complains of the ill treatment of others.

The Doctor then tells the story, that Mr. Griffith was the the inoculator, and it is fit that he also should be heard as well as the Doctor. He then breaks out again,

"Though you did not answer my letter, Rowland," (Rowland never thought it worth his while to answer an insolent letter in his life), "I conceived, knowing your genius," (I hardly think the Doctor knows any thing of my genius) "that curiosity would carry you to see this child."

"I described your dress and person to Mrs. Seyffert, mother of the child, and shewed her the print of Dr. Rowley's *cow-poxed ox-faced boy*," (another ingenious *trick* of the Doctor's for the promotion of a pestilential disease.) "I solemnly declare that I did not mention your name. She said the face of the gentleman described, was very much like that print, and from his agitation and seeming distress, she supposed he might be the father of that unhappy boy;" (so that I have not only my w——s but my bastards also,) "or the author of his misery."

"Surely, Rowland, you will not now pretend to say, that you never saw a case of small-pox after cow-pox." From the very first I never said or thought so; though I am now more than ever convinced, that after the genuine cow-pock, there are at least as few, if not even fewer, failures than after the small-pox, however communicated.

Now did ever any person beyond the confines of Bedlam, Billinsgate, or St. Giles's, meet with so much trumpery pretentions to wit, so much design and art, like people who are half deranged, and so much vulgarity of expression in a few short paragraphs?

The Doctor next tells us of *another* case, though belonging to the same family. I rather believe, had Mr. Griffith examined these cases, he might have joined his testimony with mine, that the Doctor does not at all times *stick to truth;* he would not have found them so bad as reported: but waving this, knowing nothing of the circumstance, the Doctor begins again upon me.

"Rowland, Mr. Seyffert is a poor man, with a wife and six children; he cannot afford to keep a servant or hire a nurse. I saw Mrs. Seyffert's distress, with four children in the small-pox, and her eldest daughter ill on her hands at the same time."

"The *addition* that the cow-pox had *added* to the sick, excited her indignation against THIS VILE IMPOSITION *which is practised on the poor.*" May God bless the worthy gentlemen who have been the supporters of the vaccine inoculation. I thought much better of them, especially since since I have been more connected with them in this discovery; but if they can play off such detestable tricks upon the poor, it is most terribly against them; instead of being admired for their disinterestedness, they deserve little less than the halter for their VILE IMPOSITION.

They have one consolation, however, in their favour, they practice this *vile imposition* to the great grief of the Doctor among the rich; and if the same discovery should be equally practised among the poor, and God grant it soon may, the Doctor and his brethren will fall grievously short in their fees.

We must, however, proceed a little further.

"Who do you think repaired the mischief of your pretended cow-pox security? HE, Rowland, who for several years past has scarcely been a day without prescribing for some wretched object or other labouring under the miseries of cow-pox, which you and your *blessed fraternity* have occasioned." The Doctor should not be quite so angry, when he and *his blessed fraternity* do fifty times more mischief by their variolous inoculation.

Next the Doctor's *humanity* starts up against my *cruelty* towards a family I had never seen.

"Where was this poor family to find money to pay for attendance and medicines, when the mother's hands were tied in nursing five children, and the father standing at a turnpike-gate, without going home, or even laying down on a bed for many days together, to obtain subsistence scarcely sufficient for himself. Did you put your hand in your pocket when you were there, or did you send any assistance? No, you did not. Did any other *Cowpoxer*, who went to see their children? No, none of them."

Thus ends the Doctor's story, without a scrap of truth in it. He brings me into a poor man's house by such an unaccountable method of discovery, as perhaps was never hit upon before, and where I never was in all my life; exposes me and my brother *Cowpoxers* for our cruelty, that he might tell of his own goodness on that occasion. But if he was no kinder to poor Seyffert's family, than he was to Batesford and his poor children, if they speak the truth, during the long and tiresome visits of these Doctors, we are not worse than our neighbours.

After all this, is it not time to ask what sort of public credit is to be given to such a writer for the time to come, who with so much confidence can tell such ridiculous tales and abominable falsehoods, without the least foundation from matters of fact?

Now,

Now, from the above samples may I not safely conclude, there is scarcely a doubt but that a variety of other cases brought forward in the same publication, not a little of the like art, falsehood, and exaggeration might be brought home against the Doctor, by others as well as myself. I have further to observe, that a variety of supposed cases of failure and other calamities, have been conveyed to the Doctor from the City of Bristol and its neighbourhood. Now none of these have I power to contradict, as they are unknown to me; but the correctness of these reports, speaking of them in the general; I have sufficient cause shrewdly to suspect, and that for the following reasons.

I have inoculated many hundreds in that city, and have since made every possible enquiry in my power, respecting the consequences and effects, and could not hear of any one calamitous event; while, at the same time, all the medical gentlemen of respectability in that city, as far as ever I could learn, are the universal supporters of this beneficial discovery.

Still there has been enough done, to prejudice many people against vaccination in that city, where the small-pox is frequently found to rage violently. I was told of nine children out of eleven, who were taken to the grave out of one close court only. The clamours of the antivaccinists have been the cause of all this, and infinitely more. But we are told that the variolous inoculation may prevent this evil. First, it never *has* prevented it. Next, it never *can* prevent it. Nay, it has frequently been proved, that more have been destroyed by the contagion, than have been preserved by the inoculation; and it is the highest absurdity to suppose, that the poor will ever admit such a pestilence to be introduced among them, while they are in want of almost every necessary accommodation, during the time of the existence of that wretched plague, and when the utmost care and skill are necessary to disarm it of its direful effects; and after all, as Mr. Williams, of Stockwell, has acknowledged to me (I am sorry to see his name among the number of the Doctor's correspondents,) that a sort of fatality attends their method of inoculation, especially of late, that they cannot account for, or avoid.

Mr. Williams, however, it appears, has added his quota to this low and vulgar publication, p. 249, by sending Dr. M. the story of the cow-pox failure of one John Dean and his wife, who took the disease from the cow. Has Mr. W. any evidence that it was the cow-pox? The milkers ers of cows frequently meet with similar accidents, from the pustular eruptions on the foul teats of the cow, either from the bites of venomous insects, or from other accidents; if so, the judgment he formed was more hasty than correct.

Mr. W. says, the cow-pox may be had more than once; I answer that the cow-pock may produce a local inflammation on the hands of the same subject more than once, though with very little effect indeed upon the constitution of the patient; and when lightly communicated by the lancet, instead of going through the foul chapt hands of the dairy-maid, will be productive of no consequences whatsoever. If my good friend will give me a call, I will give him a proof of this, that he cannot resist. Mr. W. also says, "that a child of Mr. Rushworth has been covered with a nasty cow-pox itch and eruptions, full of yellowish matter. He has been in that state nearly four months. This is the sixth or seventh case." Now I, who have inoculated, or seen to the inoculation of more than as many thousands, never saw any thing like it: and if he waits on Dr. Willan, the oracle on cutaneous diseases, he will teach him better. After all this, is it possible that ever Mr. Williams could inoculate with this cow-pox *venom*, and receive a fee. Alas! alas! good man! he should have remembered, that though Jehosaphat joined affinity with Ahab, yet it was much to his discredit; I should have been happy to have found my friend in better company, and engaged in defending a better cause.

Mr. Williams, however, has confessed nothing further privately to me, than what Dr. Moseley has confessed before the public at large; for he tells us, p. 146, of his being "called to three children, in different parts of the town, under inoculation: that the inoculators were men of *considerable medical reputation, but they do not understand the small-pox*." Poor gentlemen! one is sorry for their ignorance in one of the most common diseases that can be known among us. However, it seems "the consequence was, the patients were *loaded* with pustules, and not without danger." I should suppose the Doctor meant the disease in these three cases was confluent, otherwise he would not have added, "and not without danger;" so that there was more difficulty in these three cases than we Cow-poxers meet with in three thousand. The Doctor further observes, "*I flatter myself* if I had seen these children previous to the appearance of the eruptions, I could could have prevented much mischief." This is not the only instance in which the Doctor *flatters himself*.

After some similar confessions, he further adds, "even Daniel Sutton himself might meet with a *fatal* accident in his practice, where there had been no fault either in the patient, parent, or nurse. It is *a matter of surprise* to me, he has not met with *many*."

Here the Doctor appears to be *turning right about against himself*, as it is a *matter of surprise* to him, that Mr. Sutton *has not met with many fatal accidents.*

Why then, in the name of common sense, this cruel censure against these three men of *considerable medical reputation*, for *dabbling with a disease they did not understand;* when even men so great as the Doctor himself, Mr. Sutton, and others of equal great name, fame, and reputation in their variolous inoculation, may meet with *many fatal accidents* in their practice, as well as we in ours?

Now, had the Doctor and others, exemplified but one tenth part of the like candour, as it respects the evils, whether real or supposed, that may arise from the vaccine discovery, as they at times acknowledge in their variolous inoculation, especially as no science upon the earth is involved in so much perplexity as that which relates to the human frame, the controversy would not have been carried on with so much acrimony and foul abuse.

I readily acknowledge, however, that the Doctor is perfectly right in what he says, of the skill and judgment necessary to carry any patient through that dangerous disease; for after some instructions how to communicate and to treat this plague, he tells us as follows:

"The patient should be purged *every other day*, from the day of the inoculation, until the eruptions appear. The calomel alterative powder should be taken *every night*, beginning on the day of operation. This must be continued, and *in no case ought to be dispensed with*, that the habit may be under the specific influence of the mercury, on the attack of the eruptive fever.—*A strict regimen must be observed in the whole process.*"

Now all this painful and trying process upon the constitution must take place in the variolous inoculation, otherwise they who submit to it, are liable to *meet with many fatal accidents*, while we who are supposed to communicate such a *beastly*, such a *venomous* disease, need but to apply the lancet without any previous alteratives, or subsequent cathartics, on the termination of the slight disease.

After

After this, it may be time to ask what persons in their senses would venture on the variolous inoculation, unless they had Doctors as wise as Moseley, Lipscombe, Sutton, &c. to attend them, at least with their daily visits; and how is it possible that the poor can pay for such attendance, and from what quarter are they to get the money, that they may pay the apothecary for his drugs; unless they submit to be ruined in their circumstances, for the sake of preservation of their lives?

The Doctor has frequently informed the public, that the cow-pox, like the yaws, leprosy, and other similar diseases, may preserve from the small-pox for a while. So it proves; but as it happens after the constitution is entirely freed from such diseases, such a protection from the small-pox ceases to exist; and the Doctor somewhere supposes that something might be done, to render the constitution as susceptible of the small-pox, as if the cow-pox had never taken place. This would be a lucky discovery; for then the old trade would be completely restored, while the ravages of the small-pox would be as horrible, dreadful, and as profitable to these Doctors as heretofore.

Mr. Daniel Sutton it seems, comes forward boldly on this subject, and by a public advertisement, first tells us, that "Cow-pox is no security against the small-pox;" and that, "by a process *peculiar to himself, perhaps*, he is enabled in most instances, after a *certain period* from vaccination, to communicate the small-pox to such as have been deemed effectually vaccinated by the most celebrated vaccinators."—No fee will be expected in case of failure;" or, like other advertising Doctors, *No cure, no pay!*

Now, one bold challenge deserves another. He shall come and try his skill at Surry Chapel School-room; but then under this restriction, that there shall be no scratching and tearing of the arms of those who attend to be inoculated after proper vaccination, by way of making ulcers with their poisonous matter, in order that they might have to say that such sores are to be esteemed as being virtually the small-pox; and that there was no protection before such ulcers were made to exist: and there are some stories in Dr. Moseley's book, which give sufficient reason to believe, that such tricks have been played; in short, let the trial take place in a fair and honest manner, and if he, or any one else, can infect one in five hundred, or even one in a hundred, with the same sort of harmless small-pox already mentioned, as mild, or nearly so as the chicken-pox; they may then tell to all the world, what great great matters they could effect against the Jennerian discovery. But if this be a new scheme among their little party, to find out *something* after the many similar attempts which have been already made, and with so much success, this their new advertising design ought to be suspected, as being wanting in integrity, though not in art.

I fear I have gone too far, in trespassing upon the pages of your useful publication, especially as I have no legitimate right to claim the least attention on a medical subject; but alas, what is to be done with a man, who has no other mode of writing but as he opposes reason by railing, and by returning the lowest style of banter for argument and serious debate.

Let any one read him on this vaccine controversy. At a moment's thought, he dashes away; concludes that every sentiment is perfectly matured the instant it is *hatched;* then he breathes for a while, that he may publish with his comments what has been published over and over again; pleases the vulgar; disgusts the rational; and renders himself unanswerable by the very style in which he writes.

Still the mischief produced among the lower orders of people, by the publication of such vile trash, can never be exaggerated; and I am informed, that nothing has deceived the public so much, as their exposing their quackery, by way of curing cow-pox diseases, which never have existed; and which, according to the judgment of the first physicians, never can exist.

I have only for the present, to give one necessary caution respecting Mr. Lipscombe, who has done no inconsiderable mischief, by one of the boldest falsehoods that ever appeared in print, quite equal to any thing that has been recorded by Dr. Moseley himself; and so precisely is the low abusive style of this strange Doctor imitated by Mr. Lipscombe, that we may suppose the Doctor, fearing lest he might go too far, in telling so many falsehoods in the same publication, humbly requested Mr. Lipscombe's aid.—The words referred to are these.

" Rowland Hill, on the perusal of Dr. Moseley's Cow-pox Epistle, took down his cow-pox sign board under the wing of his chapel; and it is said, resolved never *to take out his lancet again,* being heartily ashamed of his vaccinating project, and determined to confine himself in future to Surry Chapel *spouting,* and the correction of the *vice* of lying, in consequence of a hint from the *Vice-suppressing Society.*"— P. 50. So

So stands this unqualified assertion; perhaps he designs something of an evasion by the words "it is said;" if so, his mean subterfuge renders him more contemptible than his notorious untruth.

It seems, however, that this report had reached down to the uttermost parts of Wales, and a letter was sent me, to enquire into the supposed fact. I should conjecture, that some *trading apothecary*, not supposing that Mr. Lipscombe, or any one else, who has a character to lose, could possibly venture upon such a vile story, was glad to retail it from place to place; and I have no doubt but that the like mischievous effects have been accomplished in other places also, by the same false declaration, which is just as true, as if he had said I had taken down the monument. I am very anxious therefore, that this public assertion may be as publicly contradicted through the medium of your Journal; and never more so than at the present hour, and for the following reason.

Corroborating proof of the general good of vaccination pours in upon me from every quarter, far beyond my most sanguine expectations, as it relates to the uncommon good of this most beneficial practice. I carefully examine almost every applicant for vaccination; I am perpetually astonished at the reports I receive of children in the same house, in the same cradle, where the disease is the most pestilential and fatal, who are protected notwithstanding; and as to subsequent diseases, I am almost tired of enquiring after them. A few diseased children, on diligent enquiry, will ever be found. But if I say there are five times more such cases to be found, after the small-pox, however communicated, to the very best of my judgment, I speak the truth. 'Though it seems almost absurd to produce fresh evidence, after what has already been laid before the public; yet there is no other method of counteracting the terrible evils of every fresh attack, but as fresh evidence is produced.

At Dursley in Gloucestershire, I had inoculated some hundreds, being but three miles from my summer's residence at Wotton Underedge. I transcribe an extract of a letter I have since then received from a shop-keeper in that place.

"Edward Birt, discharged from the Bath Hospital, catched the small-pox in that city, came to Dursley and died. Summers, a poor woman, resident in Bath, about the same time, came to Dursley to see her friends with an infant, sickened and died; a report was immediately, and by some people people industriously spread, that the woman had declared she had been inoculated for the cow-pock at Bath. I immediately applied to her relations. I found she never had been inoculated at all, but the child was inoculated last summer for the cow-pock, and is now in health; the grave-digger carried the infection to his family, and one child fell sick, which caused a vestry meeting. Dr. Fry received orders to inoculate the whole parish at 2s. 6d. each, which caused the small-pox to disappear immediately.

"I remain your very humble Servant,

*Dursley, January* 20, 1807. "JOHN HARDING."

Hence it appears, that some who were afraid of vaccination for themselves, or their offspring, were driven to it from the dangerous consequences of the small-pox; all that were vaccinated were perfectly secured, and had not vaccination protected them, the small-pox must have ravaged the town with all the dreadful consequences that universally attend it, especially among the poor.

Similar information I have also received from Wotton-Underedge. I first observe, that during the course of the summer, a woman infected with the small-pox, came into a populous close village, called Kingswood, within a mile of that town; though I had inoculated most of the people in that village before, yet immediately upon this, upwards of fifty more applied for the vaccine protection. After this, I took several children to see the woman, who was then removed a little out of the village, that they might behold the nature of the disease from which they were protected; and not a single individual was subjected to that most contagious plague.

In the town of Wotton Underedge, I know not a single family that has kept at a distance from vaccination. The consequence has been, that when two persons brought the small-pox into the town with them, they had the disease in a most dangerous manner, and one of them has never been well since; and not one person in the town was afterwards infected. A farmer and his wife, who live in a village near the town, were the only persons I could find who were averse to the vaccine protection. This last winter they had two of their children inoculated with the small-pox, one of the children recovered, the other died; it is said not of the disease, but of a locked jaw soon after the turn of the disease.

Now all the power upon the earth, could not have prevented the small-pox making its dreadful entrance into the town, town, had not the blessing of vaccination previously secured them from the attack; so that this is the second visit we have had within a few months from this pestilential disease, without any injury being received.

After all this, I conclude by observing, that Dr. Moseley, Messrs. Lipscombe, Sutton, &c. shall have my free and full consent, to send down as large a *cargo* as they please of their inoculated subjects, to go through the disease in the midst of that town, and see what may yet be the effect. They will then be at liberty to seek after all the ox-faced boys, cow-faced girls, and calf-hided children that are to be found among them; as also to find where these children are, who are supposed to be poisoned with that *beastly venom*, and which are supposed to be discovered throughout the metropolis at large.

Thankful for the great success which has hitherto attended my attempts, I cannot conclude this paper without giving a caution to slovenly inoculators. I have regularly adopted Dr. Jenner's caution, to inoculate in four places: the constitution is not always the same, and considering how slight the disease is in itself, and how slightly some people are infected, it is a matter of surprize to me that the failures are so few, and the protection so very general and secure, and a matter of infinitely greater surprize, that any should be the opposers of a discovery, that has been such an extended blessing throughout the world at large, and scarcely knows any where what opposition means, but in a nation who should be its first promoters, and warmest friends.

In addition to this paper, I meant to send some decided fresh cases of Small-pox, having occurred on different subjects; but as this paper has already swollen to a greater length than I originally intended, and as my friend Mr. Gillham has them under his care, to prepare them for the press, they shall be presented, if acceptable, in the next monthly number of your Journal.

I am, &c.

ROWLAND HILL.

P. S. Since I wrote the above, another pamphlet against vaccination has been put into my hand, written in the same vulgar abusive style, by one Ferdinand Smith Stuart, Esq.; the ridiculous artifice of a contemptible caricatured frontispiece, like the late Dr. Rowley's, evidently proves his low design. From this farrago of personal abuse, principally principally against Dr. Thornton, I transcribe but one passage.

"A child at Peckham, after being inoculated with the cow-pox, had its former natural disposition absolutely changed to the brutal, so that it ran upon all fours like a *beast*, bellowing like a *cow*, and butting with his head like a *bull*."

Now, if the printing of such shameless stuff, does not of itself destroy all the effects of such publications, nothing will.

